# Tumour inhibitory activity on pancreatic cancer by bispecific nanobody targeting PD-L1 and CXCR4

**DOI:** 10.1186/s12885-022-10165-7

**Published:** 2022-10-25

**Authors:** Shuai Hao, Shuyi Xu, Liangzhu Li, Yaxian Li, Meiqi Zhao, Junsheng Chen, Shunying Zhu, Yueqing Xie, Hua Jiang, Jianwei Zhu, Mingyuan Wu

**Affiliations:** 1 Engineering Research Center of Cell and Therapeutic Antibody, Ministry of Education, School of Pharmacy, Shanghai Jiao Tong University, 800 Dongchuan Road, 200240 Shanghai, People’s Republic of China; 2 Institute of Translational Medicine, Shanghai Jiao Tong University, 800 Dongchuan Road, 200240 Shanghai, People’s Republic of China; 3 Jecho Laboratories, Inc, 7320 Executive Way, 21704 Frederick, MD USA

**Keywords:** PD-L1, CXCR4, Antibody, Bispecific nanobody, Immunotherapy, Pancreatic cancer

## Abstract

**Background:**

Antibodies and derivative drugs targeting immune checkpoints have been approved for the treatment of several malignancies, but there are fewer responses in patients with pancreatic cancer. Here, we designed a nanobody molecule with bi-targeting on PD-L1 and CXCR4, as both targets are overexpressed in many cancer cells and play important roles in tumorigenesis. We characterized the biochemical and anti-tumour activities of the bispecific nanobodies in vitro and in vivo.

**Methods:**

A nanobody molecule was designed and constructed. The nanobody sequences targeting PD-L1 and CXCR4 were linked by the (G_4_S)_3_ flexible peptide to construct the anti-PD-L1/CXCR4 bispecific nanobody. The bispecific nanobody was expressed in *E. coli* cells and purified by affinity chromatography. The purified nanobody was biochemically characterized by mass spectrometry, Western blotting and flow cytometry to confirm the molecule and its association with both PD-L1 and CXCR4. The biological function of the nanobody and its anti-tumour effects were examined by an in vitro tumour cell-killing assay and in vivo tumour inhibition in mouse xenograft models.

**Results:**

A novel anti-PD-L1/CXCR4 bispecific nanobody was designed, constructed and characterized. The molecule specifically bound to two targets on the surface of human cancer cells and inhibited CXCL12-induced Jurkat cell migration. The bispecific nanobody increased the level of IFN-γ secreted by T-cell activation. The cytotoxicity of human peripheral blood mononuclear cells (hPBMCs) against pancreatic cancer cells was enhanced by the molecule in combination with IL-2. In a human pancreatic cancer xenograft model, the anti-PD-L1/CXCR4 nanobody markedly inhibited tumour growth and was superior to the combo-treatment by anti-PD-L1 nanobody and anti-CXCR4 nanobody or treatment with atezolizumab as a positive control. Immunofluorescence and immunohistochemical staining of xenograft tumours showed that the anti-tumour effects were associated with the inhibition of angiogenesis and the infiltration of immune cells.

**Conclusion:**

These results clearly revealed that the anti-PD-L1/CXCR4 bispecific nanobody exerted anti-tumour efficacy in vitro and inhibited tumour growth in vivo. This agent can be further developed as a therapeutic reagent to treat human pancreatic cancer by simultaneously blocking two critical targets.

**Supplementary Information:**

The online version contains supplementary material available at 10.1186/s12885-022-10165-7.

## Background

Pancreatic cancer is one of the leading causes of cancer death worldwide. Novel agents are urgently needed to treat the main pathological type, known as pancreatic ductal carcinoma (PDAC). Due to a lack of early diagnosis and effective treatments, even if patients undergo complete resection and receive chemotherapy and radiotherapy, the 5-year survival rate is less than 26% [1]. Great effort has been dedicated to understanding the mechanisms of this disease [2–5]. PDAC remains difficult to treat due to the high heterogeneity, suggesting that no single targeted therapy will work for all PDAC patients [6].

Immune-based therapeutic strategies have been promising in the treatment of cancers [7–9]. In recent decades, one of the major breakthroughs in cancer immunotherapy has been the discovery of immune checkpoint molecules, such as cytotoxic T-lymphocyte-associated protein 4 (CTLA-4), programmed cell death-1 (PD-1) and its ligand [programmed death-ligand 1 (PD-L1)]. Then, anti-CTLA-4, anti-PD-1 and anti-PD-L1 monoclonal antibodies and other immune checkpoint inhibitors have been markedly successful in treating diverse cancer patients with melanoma, non–small-cell lung cancer, and ovarian cancer [10–12]. However, PDAC is unfortunately the exception because of its unique tumour microenvironment (TME) [12, 13].

The PDAC microenvironment is characterized by increased desmoplasia and the accumulation of immunosuppressive cells, which play determinant roles during tumour development [14]. Immune suppression in the tumour microenvironment (TME) can be regulated by secreted cytokine- and chemokine-mediated signalling pathways, which are known to protect tumour cells from immune attack. Chemokine (C-X-C motif) ligand 12 (CXCL12), also known as stromal cell-derived factor 1 (SDF-1), which is produced by carcinoma-associated fibroblasts (CAFs), stimulates pancreatic cancer cell proliferation, recruits regulatory T cells (Tregs) and promotes desmoplastic alterations in the surrounding stroma through the CXCR4/CXCL12 axis [15–17]. Thus, CAFs create a collagen-rich barrier to prevent cytotoxic T lymphocytes (CTLs) from entering the tumour site. Administering a CXCR4 antagonist to PDAC-bearing mice results in the rapid accumulation of CTLs, the arrest of tumour growth, and increased tumour sensitivity to the anti-PD-L1 antibody [15].

Nanobodies (Nbs), also named variable domain of the heavy chain of heavy-chain antibody (VHH), have been developed as an alternative to conventional antibodies and appear to have many advantages in therapeutic applications. These factors are relatively small in size, with advantages in tissue penetration and cavity binding, allowing them to cross the blood-brain barrier and accumulate in targeted organs such as brain diseases and pulmonary and gastrointestinal tract tumours [18, 19]. Moreover, the high affinities, better stability, possibility of formatting or multimerization provide this group of molecules with unique properties and druggability [20]. In 2018, the first nanobody drug caplacizumab was approved by the European Medical Agent for the treatment of adult-acquired thrombotic thrombocytopenic purpura (aTTP) patients [21]. Several additional nanobodies have been in various phases of clinical development [22, 23].

Based on cumulative research on cancer immunotherapy in our laboratory, we have established a symmetric research program from designing bispecific antibodies to comprehensively characterize a wide spectrum of tumours in vitro and in vivo [24–28]. Here, we report recent progress on the design and characterization of a novel bispecific nanobody targeting PD-L1 and CXCR4. The anti-tumour effects of the anti-PD-L1/CXCR4 nanobody were evaluated in tumour cell-killing assays in vitro and in an in vivo xenograft mouse model of PDAC.

## Materials and methods

### Reagents and cell culture

FITC-labelled anti-His monoclonal antibodies (mAbs) were purchased from Abcam Company (Cambridge, UK). Mouse anti-His tag mAbs were purchased from Proteintech Group, Inc. (Wuhan, China). Goat anti-mouse IgG-HRP was purchased from Yeasen Biotech Co., Ltd. (Shanghai, China). APC-labelled rabbit anti-human PD-L1 mAbs were purchased from Sinobiological, Inc. (Beijing, China). A bispecific anti-human CD3/CD171 antibody was produced in-house. Atezolizumab was purchased from Selleck Chemical Company (Texas, USA). Recombinant human interleukin-2 (IL-2) was purchased from Huaxin Biotech Co., Ltd. (Shanghai, China). Human CXCL12 (hCXCL12, SDF-1α) was purchased from PeproTech (New Jersey, USA). The human interferon-γ (IFN-γ) ELISA kit was from R&D System (Minnesota, USA). Ficoll-Paque® PLUS was purchased from GE Healthcare (Wisconsin, USA).

Human peripheral blood mononuclear cells (hPBMCs) were provided by healthy volunteers (Changhai Hospital, Shanghai, China). Human cancer cell lines (Panc-1, AsPC-1, U251-MG and Jurkat cells) were purchased from the Cell Bank of the Chinese Academy of Sciences (Shanghai, China). Panc-1, AsPC-1 and Jurkat cells were cultured in RPMI 1640 medium with 10% fetal bovine serum (FBS). U251-MG cells were cultured in DMEM supplemented with 10% FBS.

### Plasmid construction

The anti-human PD-L1 VHH sequence used in this paper was previously reported [29], and the sequence of anti-human CXCR4 bivalent VHH was obtained from an issued patent (International Publication Number WO2011/161266A1). The two fragments were connected by a (G4S)_3_ linker to assemble the anti-PD-L1/CXCR4 bispecific nanobody (BsNb PX4). The sequence of the anti-PD-L1/CXCR4 nanobody with the C-terminal 6×His-tag was synthesized by GenScript® ProBIO (Nanjing, China) and cloned into the pET-22b (+) vectors. The pET-22b (+) vectors and targeted fragments were digested by Nco1 and EcoR1 restriction enzyme, with the ligation reaction using T4 DNA Ligase.

### Expression and purification

The recombinant BsNb PX4 plasmid was transferred into *Escherichia coli (E. coli)* BL21 (DE3). Positive transformants were collected and cultured in Terrific Broth (TB) medium at 37 °C, and then the culture was induced with 0.1 mM isopropyl-β-D-thiogalactoside (IPTG). After 18 h of induction at 25 °C, the cells were collected by centrifugation, resuspended in phosphate buffered saline (PBS), and lysed using a high-pressure homogenizer. The cell lysate was centrifuged at 12,000 r/min at 4 °C for 30 min to remove cell debris, and the supernatant was applied to a HisTrap affinity column (GE Healthcare, Wisconsin, USA). Then, the protein of interest was separated by step elution with 2 M imidazole, and the fractions containing the target protein were pooled and concentrated by using ultrafiltration tubes with a molecular weight cut-off (MWCO) of 10 kDa (Millipore, Tennessee, USA). The proteins were analysed by SDS-PAGE and transferred to a PVDF membrane. The blots were incubated with the mouse anti-His tag mAbs, followed by incubation with goat anti-mouse IgG-HRP. The protein bands were detected by an ECL Ultra Kit (NCM Biotech, Suzhou, China).

### Q-TOF/LC‒MS

The purified bispecific nanobody (1 mg/mL) was concentrated in a 0.05 mmol/L NH_4_HCO_3_ solution. An ultrahigh pressure liquid system (ACQUITY I-Class) was connected to a VION IMS-Q-TOF (Waters, Massachusetts, USA). The MassPREP (Waters, Massachusetts, USA) desalting column was used for UPLC with a controlled column temperature of 60 ℃. Mobile phase A was a 0.1% formic acid-water solution, and mobile phase B was a 0.1% formic acid-acetonitrile solution. The system was preequilibrated with 95% mobile phase A, and then 0.5 µg of the bispecific nanobody was injected and eluted with a gradient at a flow rate of 0.2 mL/min. The mass spectrometry conditions were as follows: capillary voltage, 2.0 kV; sampling cone voltage, 60 V; ion source temperature, 115 °C; desolvent gas temperature, 500 °C; and desolvent gas flow rate, 900 L/min. Data were collected by UNIFI 1.8, and mass (Da)-intensity was obtained by deconvolution analysis.

### Flow Cytometric Analysis

The nanobodies were tested for binding to Jurkat, U251-MG, AsPC-1, and Panc-1 cells. The cells (1 × 10^5^ per well) were incubated with BsNb PX4, anti-CXCR4 nanobodies, or anti-PD-L1 nanobodies (1 µg/mL). FITC-labelled anti-His secondary mAbs were used. The samples were washed twice with FACS buffer (ice-cold PBS containing 2% FBS), and data were acquired on a CytoFLEX cytometer (Beckman Coulter, California, USA).

PD-L1 and CD171 double-positive U251-MG cells were labelled with CFSE green fluorescent dye (Invitrogen, California, USA), and CXCR4 and CD3 double-positive Jurkat cells were labelled with PKH26 red fluorescent dye (Sigma, Missouri, USA) according to the manufacturer’s protocols. The cells were resuspended and washed three times with FACS buffer. The labelled cells were mixed at an equal ratio (U251-MG cells to Jurkat cells) and then treated with BsNb PX4 for 30 min at 4 °C. The bispecific anti-CD3/CD171 antibody was included as a positive control, and the anti-CXCR4 nanobody was included as a negative control. Flow cytometric analysis was performed to detect CFSE + PKH26 + cells.

### Chemotaxis Assay

Cell migration was evaluated using a Transwell chamber with 5 μm pore filters (Corning Inc., New York, USA). Jurkat cells (2.5 × 10^5^ per well) were added to the upper chambers, and 20 µL of 200 ng/µL hCXCL12 was added to the lower chamber. Jurkat cells were allowed to migrate towards hCXCL12 for 4 h at 37 °C in 5% CO_2_. To examine antagonistic properties, 0.08 µM BsNb PX4 or the anti-CXCR4 nanobody was added to the upper chamber. To determine the half-maximal inhibitory concentration (IC_50_), different concentrations of BsNb PX4 were added to the upper chamber. The migrated cells in the lower chamber were collected and quantified by an automated cell counter (Countstar, Shanghai, China).

### Analysis of the production of IFN-γ by ELISA

hPBMCs from a healthy volunteer were isolated by Ficoll-Paque® PLUS density gradient centrifugation. T cells were sorted and purified from hPBMCs by using CD3 microbeads (Miltenyi Biotec, Köln, Germany). T cells (6 × 10^4^) were cultured in 96-well plates for 24 h at 37 °C in RPMI 1640 supplemented with 10% FBS in the presence of 50 ng/mL anti-CD3 antibodies and 50 U/mL interleukin-2. After the T cells were activated, 0.3 nM BsNb PX4 and anti-PD-L1 nanobodies were added. Supernatants were collected after 24 h, 48 and 72 h, and the level of IFN-γ was determined by ELISA.

### Detection of cell viability and proliferation

To determine the inhibitory effects of the nanobodies, 5 × 10^3^ tumour cells were seeded in 96-well plates and incubated with 0.00001 µM, 0.001 µM and 0.1 µM BsNb PX4 for three days. AsPC-1 cells were plated (5 × 10^3^ per well) in 96-well plates with or without 200 ng/µL hCXCR12. After 4 h at 37 °C, BsNb PX4 and anti-CXCR4 nanobodies were added to a final concentration of 0.1 µM. Cell viability and proliferation were measured using a Cell Counting Kit-8 assay (Dojindo, Tokyo, Japan).

### ***In vitro*** cytotoxicity assays

Panc-1 or AsPC-1 cells were seeded at 5 × 10^3^ per well overnight in RPMI-1640 medium with 10% FBS before hPBMCs were added at a 10:1 E/T ratio, followed by incubation for 24 h with IL-2 (100 IU/mL). Then, the mixture was cultured for an additional 48 h in the presence of BsNb PX4 (0.5 µM). To examine tumour cell lysis, the release of lactate dehydrogenase (LDH) in the coculture supernatants was measured by a CytoTox 96® Non-Radioactive Cytotoxicity Assay Kit (Promega, Wisconsin, USA). The percentage of cytotoxicity was calculated as follows: cytotoxicity% = (experimental lysis–spontaneous effector lysis − spontaneous target lysis)/(maximum target lysis − spontaneous target lysis) × 100%.

### ***In vivo*** activity of bispecific nanobody with hPBMCs

Female NOD/SCID mice aged 6–7 weeks were purchased from Beijing Vital River Laboratory Animal Technology Co., Ltd. (Beijing, China) and housed in a specific pathogen-free room under controlled temperature and humidity conditions. All animal experiments were performed in accordance with the guidelines of the Institutional Animal Care and Use Committee of Shanghai Jiao Tong University. The NOD/SCID mice were subcutaneously injected with 2 × 10^6^ AsPC-1 cells. When the mice bearing AsPC-1 tumours ranging from 50 to100 mm^3^ were established, 2 × 10^6^ human PBMCs were injected into the tail vein. AsPC-1 cells inoculations and hPBMCs injections were performed under anesthesia with 2% isoflurane inhalation. The mice were randomly divided into four treatment groups: PBS, atezolizumab, BsNb PX4, combination of anti-CXCR4 nanobody and anti-PD-L1 nanobody. The first treatment was administered 1 day after the inoculation of hPBMCs. BsNb PX4 (0.3 mg/kg) or combination of the two nanobodies (anti-CXCR4-VHH at 0.3 mg/kg; anti-PD-L1-VHH at 0.3 mg/kg) was injected intraperitoneally (i.p.) every two days for a total of five times. The positive control drug atezolizumab was administered i.p. at a dose of 3 mg/kg every five days for a total of two times. The tumour volumes were measured every three days and calculated as follows: 0.5×length×width^2^.

### Immunohistochemistry (IHC) and immunofluorescence (IF) staining

All mice were sacrificed by cervical dislocation under anesthesia with 2% isoflurane inhalation before the tumour volume reached 2000 mm^3^. The tumour grafts were removed, embedded in paraffin and sectioned into 3 μm sections. IHC was performed according to standard protocols. Antigen retrieval was achieved by heating the sections in 0.1 M Tris-HCl buffer (pH 9.0) at 98 °C for 15 min. Endogenous peroxidase was blocked with 3% H_2_O_2_ in PBS for 20 min. Subsequently, the sections were blocked with normal goat serum, followed by incubation with primary antibodies (mouse anti-His tag mAbs) at a 1:200 dilution overnight at 4 °C. The sections were stained using a polymer HRP detection system (DAKO, California, USA). The images were captured using an XSP-C204 (COIC, Chongqing, China).

For IF staining, the sections were blocked with 5% BSA for 1 h at room temperature and incubated with CD31 and αSMA antibodies (CST, Massachusetts, USA) for 2 h in the dark. Appropriate DyLight® fluorochrome-conjugated secondary antibodies (Earthox, Massachusetts, USA) were used at 1:200 dilutions for 1 h at 37 °C. After being immunolabelled, the sections were washed and stained with DAPI (Sigma, Mississippi, USA) for 10 min at room temperature. The images were visualized under a NIKON ECLIPSE C1 microscope (NIKON, Tokyo, Japan).

### Statistical analysis

All quantification data are summarized as the mean ± standard deviation (SD) of at least three biological replicates of each experiment. GraphPad Prism 8.0 (GraphPad Software Inc., CA, USA) was used to create the graphs. Standard *t* tests were used to compare 2 groups, and one-way ANOVA was used to compare more than 2 groups. For all statistical comparisons, *P* < 0.05 indicated a statistically significant difference.

## Results

### Molecular design, generation, and characterization of anti-PD-L1/CXCR4 bispecific nanobodies

**Fig. 1:**
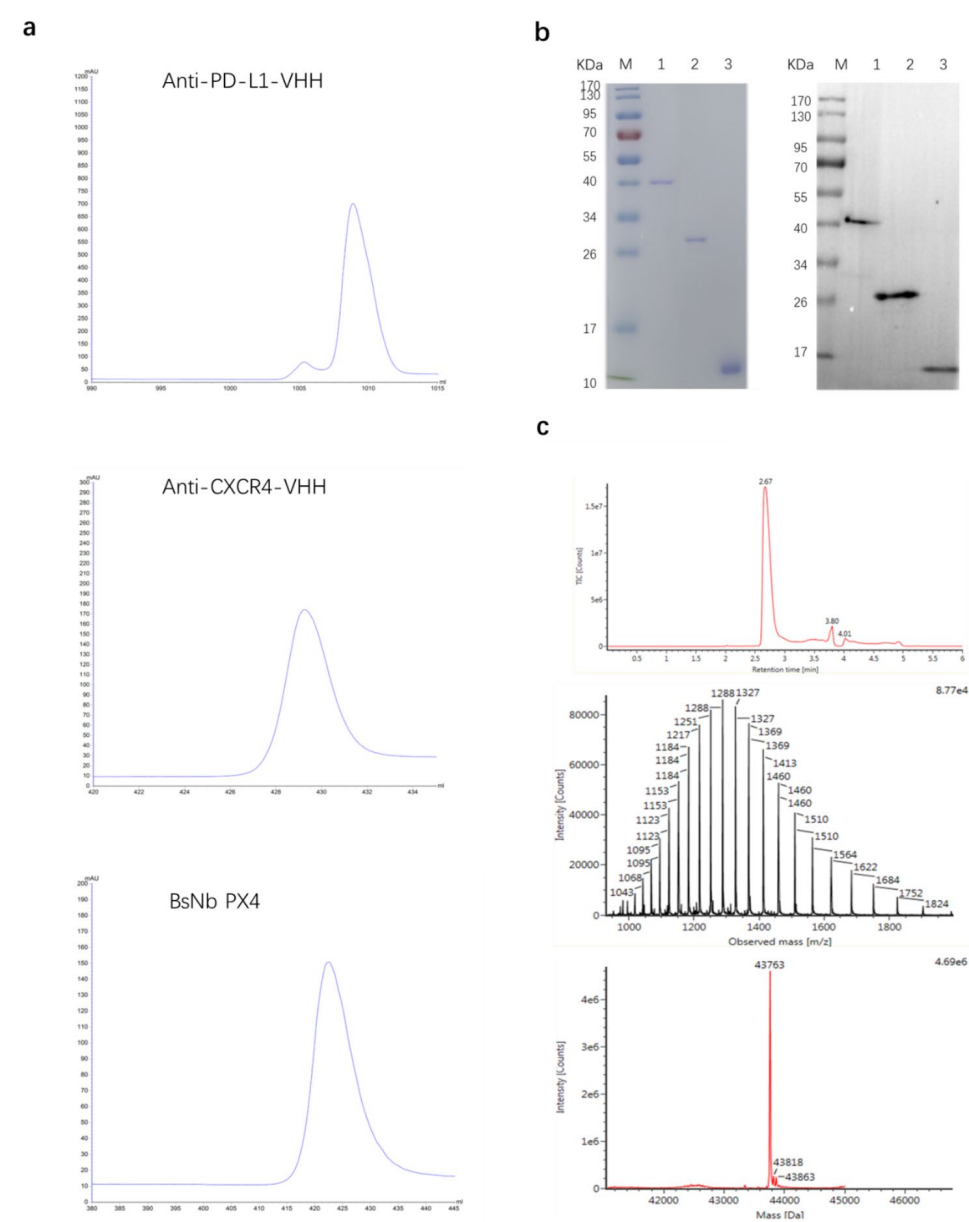
Purification and identification of the nanobodies. **a** Purification of the nanobodies using a His-tag affinity chromatography column on an ÄKTA Start System. **b** SDS‒PAGE and Western blot analysis of the nanobodies (the cropped gel/blot are displayed here for better layout, and the full-length gels/blot are shown in Supplementary Fig. 1). Lane M, low molecular weight (MW) protein markers. Lane 1, BsNb PX4; Lane 2, Nb CXCR4; Lane 3, Nb PD-L1. **c** Q-TOF/LC‒MS to identify the complete MW of BsNb PX4

We designed a nanobody molecule targeting PD-L1 and CXCR4 with anti-PD-L1 VHH and two anti-CXCR4 VHH fragments. Both antibodies were bridged by the short linker peptide (G_4_S)_3_. The expression vectors (described in Materials and Methods) were transformed into the *E. coli* BL21(DE3) strain. Nanobody expression was induced by 0.1 mM IPTG after the cell density reached more than OD_600_ 0.6. The nanobodies were purified through a HisTrap Ni^2+^ chelating column (Fig. 1a). The expected molecular sizes of the anti-CXCR4 nanobody (Nb CXCR4), the anti-PD-L1 nanobody (Nb PD-L1) and BsNb PX4 were approximately 29 kDa, 15 kDa, and 44 kDa, respectively (Fig. 1b). The identity of the purified proteins was further confirmed by mass spectrometry analysis to confirm the calculated mass of BsNb PX4 (Fig. 1c).

The results showed that the bispecific anti-PD-L1/CXCR4 nanobody (BsNb PX4) purity was greater than 95%, and the characterization was consistent with our design expectations, which met the requirements for in vitro and in vivo assays.

### Association of the anti-PD-L1/CXCR4 bispecific nanobody with its targets

**Fig. 2:**
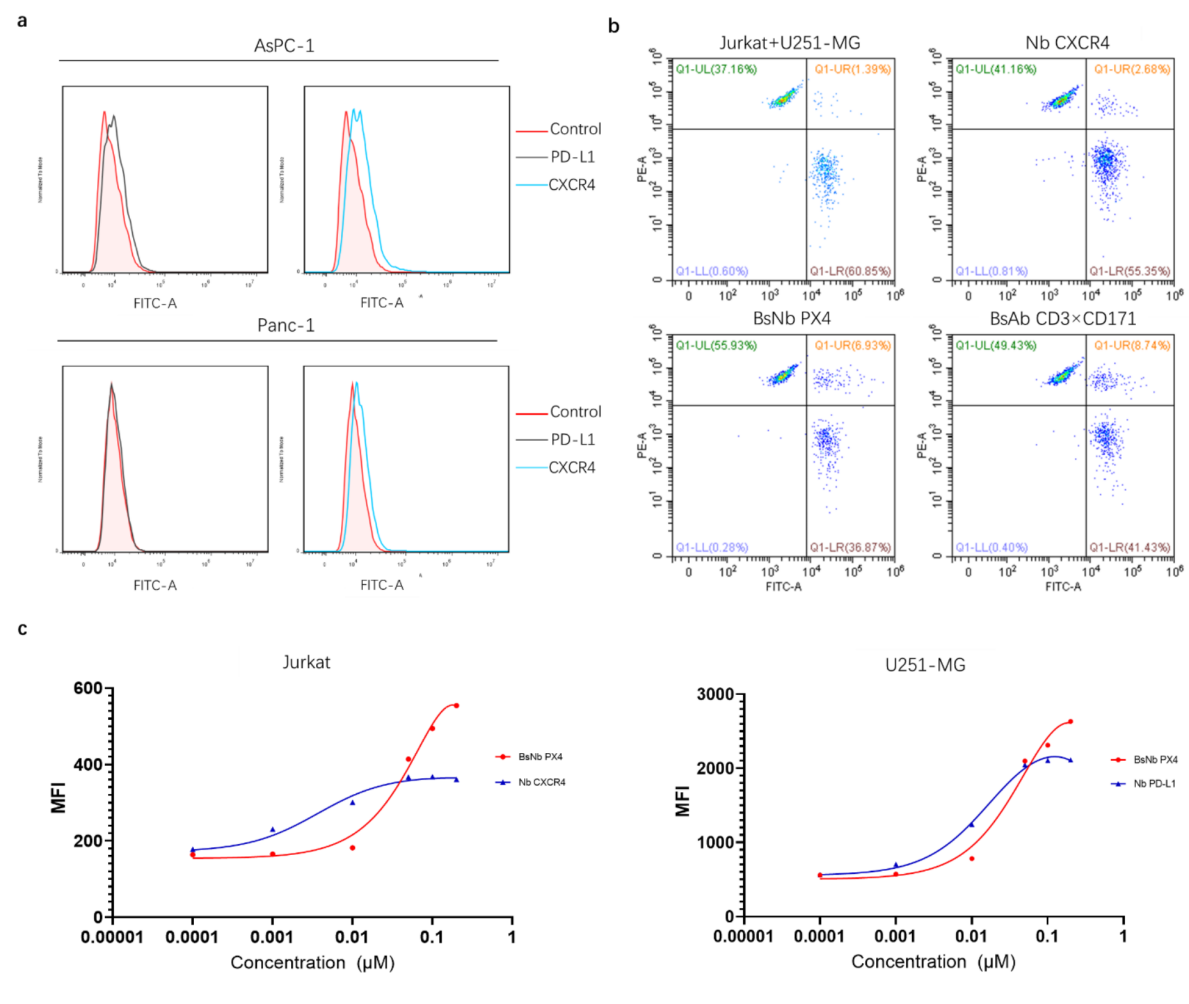
Binding properties of the nanobodies, as analysed by flow cytometry. **a** AsPC-1 and Panc-1 cells were stained with parental Nb PD-L1 and Nb CXCR4. **b** Analysis of BsNb PX4-induced cellular clustering when U251-MG cells and Jurkat cells were mixed at a 1:1 ratio. Quantification of the flow cytometry results showing the percentage of events that represent cellular clusters. **c** Curve showing BsNb PX4, Nb PD-L1, Nb CXCR4 binding with PD-L1^+^U251-MG or CXCR4^+^ Jurkat cells

To evaluate the binding properties of these nanobodies, we examined their associations with cell surface antigens by flow cytometry. The relative expression levels of PD-L1 and CXCR4 on the surface of human pancreatic cancer cell lines (AsPC-1 and Panc-1) were first evaluated. The results showed that both antigens were expressed on AsPC-1 cells, and only CXCR4 was expressed on the surface of Panc-1 cells (Fig. 2a). The increased proportion of cellular clusters in the double-positive area (Q1-UR) preliminarily verified the recruitment effect of BsNb PX4 (Fig. 2b). For PD-L1^+^U251-MG cells and CXCR4^+^ Jurkat cells, the binding of BsNb PX4 (KD = 73.5 nM) was slightly weaker than that of Nb PD-L1 (KD = 19.1 nM; ~4-fold), while compared with Nb CXCR4 (KD = 3.7 nM; ~50-fold), BsNb PX4 showed a significant decrease in affinity (KD = 185 nM) (Fig. 2c). Altogether, our results indicated that bispecific nanobody retained specifically binding to the surface antigens PD-L1 and CXCR4 as the parental nanobodies.

### BsNb PX4 inhibited CXCL12-induced chemotaxis and promoted IFN-γ production

**Fig. 3:**
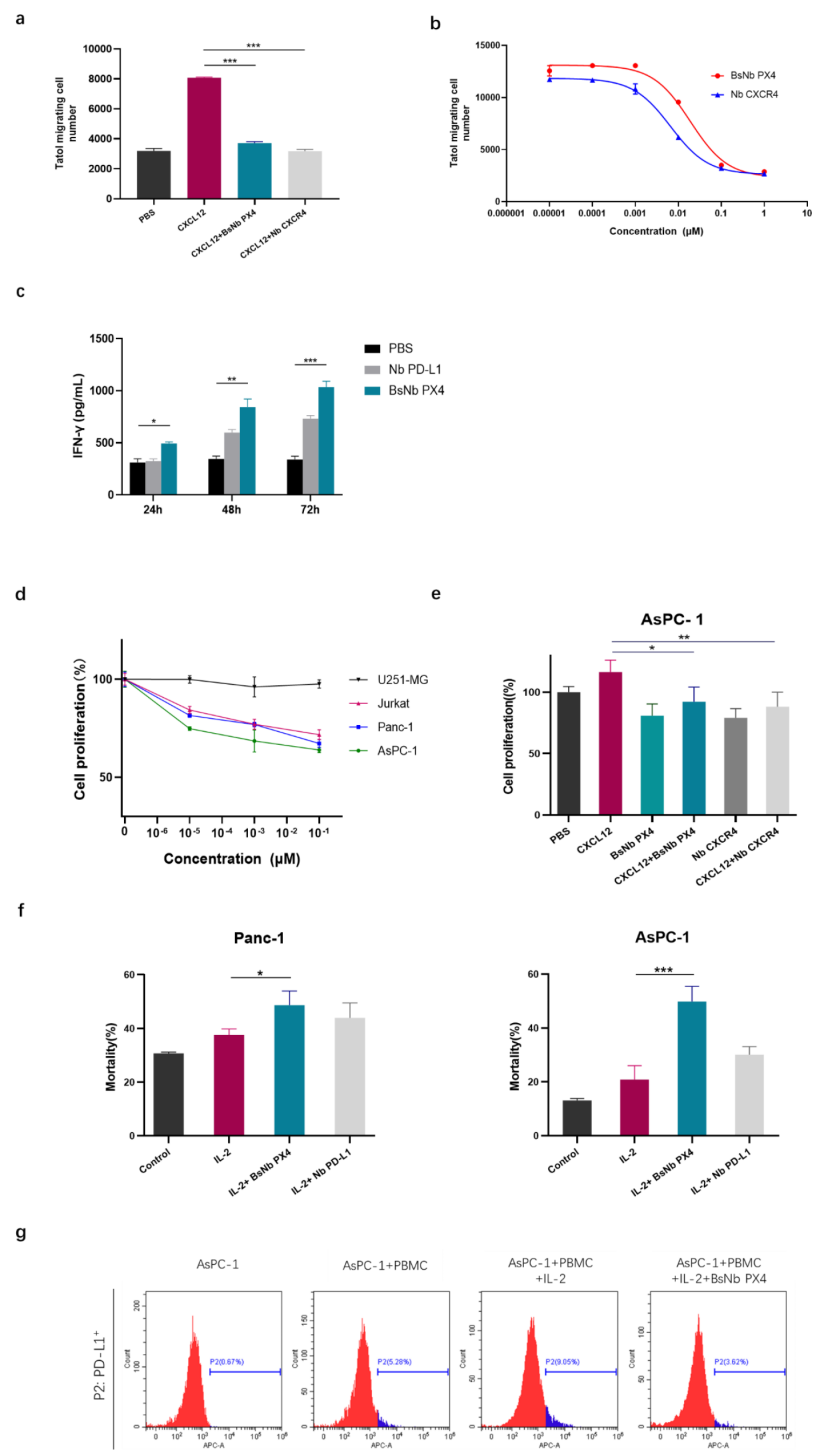
Bioactivity analysis of bispecific nanobodies in vitro. **a** Jurkat cell chemotaxis was inhibited by BsNb PX4 and Nb CXCR4. **b** The half-maximal inhibitory concentrations of BsNb PX4 and Nb CXCR4. **c** The levels of IFN-γ were measured by ELISA. **d** Inhibition of cancer cell proliferation induced by BsNb PX4. **e** Inhibition of AsPC-1 cell proliferation induced by BsNb PX4 in the presence of CXCL12. **f** Cytotoxicity of PBMCs against tumour cells treated with BsNb PX4 and IL-2 in vitro. Control group: hPBMCs incubated with tumour cells for 96 h; IL-2 group: 100 IU/mL IL-2 was added to wells after 24 h of hPBMC and tumour cell coculture, then the mixture was cultured for 72 h; IL-2 + BsNb (or IL-2 + Nb PD-L1) group: hPBMCs were precultured with tumour cells for 24 h, followed by incubation with IL-2 for 24 h, and then the mixture was cultured for 48 h in the presence of BsNb PX4 (or Nb PD-L1). **g** Changes in PD-L1 expression levels in AsPC-1 cells were analysed by flow cytometry. (**a-f**) The data are shown as the mean ± S.D. (n = 3). *P < 0.05, ** *P* < 0.01, ***P < 0.001

To examine the antagonistic effect of the nanobodies on chemotaxis, the migration of Jurkat cells was evaluated by Transwell chamber assays. Compared with that in the negative group, the nanobodies clearly inhibited the migration of Jurkat cells induced by hCXCL12; however, no significant differences between BsNb PX4 and Nb CXCR4 were observed (Fig. 3a). The IC_50_ of BsNb PX4 was approximately 18.99 nM for Jurkat cell mobility (Fig. 3b).

To investigate whether BsNb PX4 blocks the negative signalling that suppresses T-cell function, we measured the IFN-γ secretion by activated T cells. After being stimulated with anti-CD3 antibody and IL-2, T cells slightly increased CD69 levels, produced the cytokine IFN-γ, and the expression of PD-L1 was also elevated. The immune checkpoints PD-1/PD-L1 were triggered to attenuate the activity of T cells. Nb PD-L1 and BsNb PX4 could relieve the negative regulation and promote the persistence of activated T cells. At 48 and 72 h, the level of IFN-γ in the BsNb PX4 group was obviously elevated compared with that in the control group (Fig. 3c).

### BsNb PX4 decreased tumour cell viability by enhancing the cytotoxicity of hPBMCs

To determine whether the inhibitory effect of BsNb PX4 on tumour cells correlated with the expression of CXCR4, we examined the proliferation of four different cancer cell lines using CCK-8 assays. BsNb PX4 decreased the viability of CXCR4-expressing tumour cell lines, including Jurkat cells, AsPC-1 cells and Panc-1 cells, in a dose-dependent manner. We also found that the inhibitory effects of BsNb PX4 on the human pancreatic cancer cell AsPC-1 were superior to those on other cell lines (Fig. 3d). In addition, pretreatment of AsPC-1 cells with BsNb PX4 or Nb CXCR4 resulted in a negative impact on cell proliferation, which indicated that the inhibitory effect of BsNb PX4 on tumour cells might involve blocking the CXCL12/CXCR4 signalling pathway (Fig. 3e).

The cytotoxicity of hPBMCs on pancreatic cancer cell lines was examined with an LDH release assay. Furthermore, hPBMCs were incubated with target cells, PANC cells or AsPC-1 cells at a ratio of 10:1. Treatment with BsNb PX4 combined with IL-2 considerably enhanced the cytotoxicity of hPBMCs against pancreatic cancer cell lines compared with the effect of IL-2 alone. In AsPC-1 cells, combined treatment with BsNb PX4 and IL-2 was more effective than that of Nb PD-L1 and IL-2 (49.8 ± 5.67% vs. 30.1 ± 2.98% on AsPC-1 cells; 48.6 ± 5.27% vs. 43.9 ± 5.60% on Panc-1 cells) (Fig. 3f). We further analysed the expression of PD-L1 on AsPC-1 cells. When the cells were incubated with hPBMCs, the level of PD-L1 detected by flow cytometry (APC Rabbit Anti-Human PD-L1) was markedly increased from 0.67 to 5.28%, particularly in the presence of IL-2, and reached 9.06%. The measured PD-L1 level on AsPC-1 cells was down to 3.62% in the IL-2 + BsNb PX4 treatment group, and that may be due to inaccessibility of PD-L1 epitope already bound BsNb PX4. (Fig. 3 g).

### BsNb PX4 inhibited the growth of human pancreatic cancer cells implanted in NOD/SCID mice

**Fig. 4:**
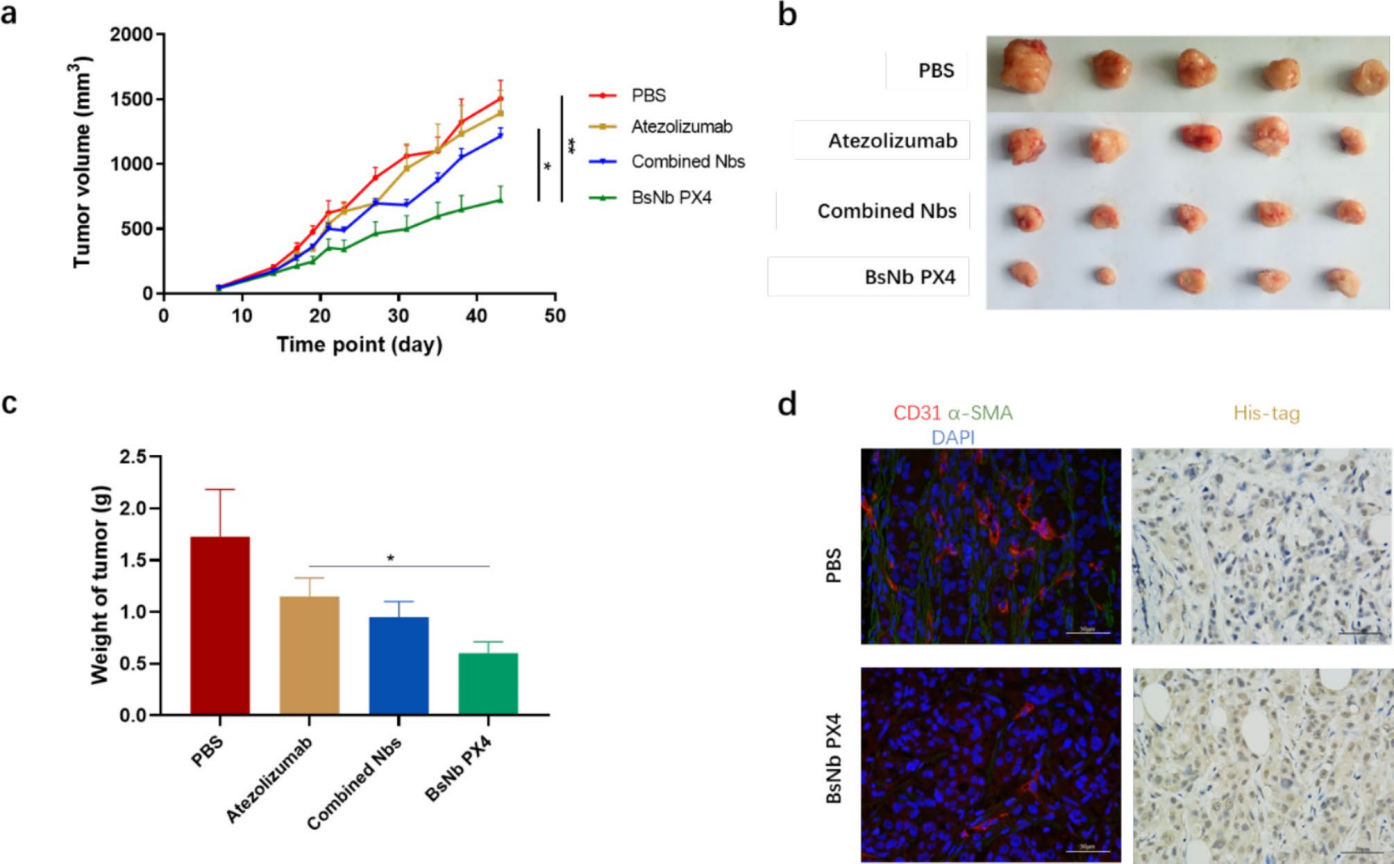
Therapeutic effect of the bispecific nanobody in vivo. **a** Tumour growth curve of the AsPC-1 mouse xenograft model in the different treatment groups (N = 5/group). Tumour volumes were measured by Vernier callipers every 3 days. A total of 2 × 10^6^ AsPC-1 cells were injected into the right flank of each mouse, and 2 × 10^6^ unstimulated PBMCs were injected into the caudal vein after the tumours reached approximately 100 mm^3^ in size. The mice were administered with PBS, 3 mg/kg atezolizumab and the two nanobodies (0.3 mg/kg Nb CXCR4 and 0.3 mg/kg Nb PD-L1) or 0.3 mg/kg BsNb PX4. **b** All tumour masses were photographed. **c** Weight of the tumour masses. **d** Tumour sections were stained with anti-CD31, anti-αSMA and anti-His-tag mAbs. (**a-c**) The data are presented as the mean ± S.D. (n = 5); *P < 0.05, ** *P* < 0.01, ***P < 0.001. “Combined Nbs” represents the co-treatment group of Nb CXCR4 and Nb PD-L1

The human pancreatic cancer cell AsPC-1 xenograft mouse model was used to validate the anti-tumour potential of the bispecific nanobody in vivo. Atezolizumab, a humanized monoclonal antibody of the IgG1 isotype against PD-L1, was selected as a positive control. The curve showed that combination treatment with BsNb PX4 and PBMCs significantly inhibited tumour growth compared with PBS as a placebo (Fig. 4a). All tumours were collected and weighed at the end of the experiment. The tumour volumes in the BsNb PX4 group were smaller than those in the atezolizumab group or the combination treatment group, including Nb PD-L1 and Nb CXCR4 (Fig. 4b). The tumour weight in the BsNb PX4 group was markedly less than that in the other groups (Fig. 4c). In addition, immunohistochemical analysis confirmed that the bispecific nanobody could effectively penetrate the tumour tissue. Immunofluorescence staining was performed to assess tumour angiogenesis and fibrogenesis with anti-CD31 and anti-αSMA antibodies. The results demonstrated that BsNb PX4 reduced tumour neovascularization and desmoplasia compared with those in the negative control group (Fig. 4d).

## Discussion

It is now clear that cancers use negative signal pathways as the major mechanism of immune evasion. Most pancreatic ductal adenocarcinomas (PDACs) are characterized by the invasion of immune suppressor cells, such as myeloid-derived suppressor cells (MDSCs), Tregs and tumour-associated macrophages (TAMs). MDSCs are able to inhibit CTLs, leading to the failure of efficient anti-tumour responses [30–32]. Moreover, MDSCs are also able to induce the proliferation of Tregs through the secretion of transforming growth factor-beta (TGF-β) and PD-L1 expression at the tumour site. The level of infiltrating Tregs in PD-L1-positive tumour sections is higher than that in PD-L1-negative tumours [33–35]. The PD-L1/PD-1 interaction leads to the suppression of T-cell activation and self-tolerance. Thus, PDAC establishes an immunosuppressive tumour microenvironment. Antibodies against PD-1 and PD-L1 have been used as cancer immunotherapy. However, single PD-1/PD-L1 immune checkpoint blockade has a poor effect on PDAC patients [12, 36].

The unique PDAC microenvironment is also composed of the surrounding extracellular matrix (ECM), hyaluronan, and stromal cells. Carcinoma-associated fibroblasts (CAFs) play an important role in maintaining the stroma. Activated CAFs produce FAP, TGF-β and platelet-derived growth factor (PDGF), favouring tumour cell growth and invasion, extracellular matrix deposition, and angiogenesis within the tumour matrix [37]. These components together develop a desmoplastic stroma, which prevents conventional chemotherapeutic drugs, such as gemcitabine from penetrating, and results in drug resistance in PDAC [38, 39]. Stromal depletion may increase the efficacy of chemotherapeutic drugs and irradiation by improving drug and oxygen delivery [40]. CAFs are also the primary source of CXCL12, which interacts with the receptor CXCR4 to stimulate pancreatic cancer cell proliferation, regulating the infiltration of CTLs and the recruitment of Tregs [15–17]. Furthermore, the CXCR4 expressed on the surface of cancer cells attracts CAFs to the tumour environment, which in turn attracts CXCR4-positive bone marrow-derived progenitor cells and participates in angiogenesis [16, 41]. Modulation of the CXCL12/CXCR4 axis in ovarian cancer has multimodal effects on pathogenesis and is associated with induction of anti-tumour immunity [17]. Downregulation of CXCL12/CXCR4 signalling enhanced sensitivity to chemotherapy and impaired tumour growth in a xenograft mouse model of hepatocellular carcinoma [42]. BL-8040, a small molecule inhibitor of CXCR4 has been shown to expand the benefits of chemotherapy in combination with pembrolizumab on pancreatic cancer [43].

Due to the immunosuppressive tumour environment and dense desmoplasia, therapeutic regimens are hindered in clinical PDAC patients. Compared with traditional antibodies, nanobodies can easily enter the tumour microenvironment due to their small size. The anti‑PD‑L1 nanobody binds to the PD‑L1 molecule with high affinity and enhances the anti-tumour immune response by blocking PD-1/PD-L1 signalling and promoting IFN-γ secretion [29, 44]. Moreover, envafolimab (KN035) is the first PD-L1 nanobody to enter clinical development [45]. Based on the results of a pivotal phase II trial (ClinicalTrials.gov, NCT03667170), envafolimab was approved by the National Food and Drug Administration of China in November 2021, for the treatment of adult patients with previously-treated microsatellite instability-high (MSI-H) or deficient MisMatch Repair (dMMR) advanced solid tumours [46]. In addition, due to the unique structure of nanobodies, envafolimab is administered by subcutaneous injection (SC), which can improve patient compliance. Phase I clinical data shows that envafolimab has favourable safety and pharmacokinetic characteristics [47]. On the other hand, nanobody ALX-0651, targeting two different epitopes of CXCR4 against GPCRs, has been discontinued due to the lack of better clinical efficacy compared to standard therapies [48]. And then, the anti-CXCR4 nanobody-Fc competitively inhibits CXCR4-mediated HIV entry and blocks CXCL12-induced chemotaxis as well as anti-CXCR4 nanobody [49, 50].

The targets PD-L1 and CXCR4 are expressed in various cancers and tumour microenvironments. We confirmed that the level of PD-L1 on AsPC-1 pancreatic cancer cells was upregulated when the cells were cocultured with PBMCs and IL-2. BsNb PX4 antagonized the PD-1/PD-L1 negative signal and strengthened the cytotoxicity of T cells against AsPC-1 cells through the secretion of IFN-γ. In a xenograft mouse model of human pancreatic cancer, we validated the anti-tumour effects of BsNb PX4 combined with hPBMCs, which were superior to the effects of two nanobodies. Blocking the CXCR4/CXCL12 signalling axis promotes T-cell accumulation and acts synergistically with anti-PD-L1 to cause cancer regression [15]. Furthermore, BsNb PX4 reduced αSMA expression of tumour tissue. PDAC is usually characterized by a prominent desmoplastic stroma that is induced partially composed of αSMA [51]. Therefore, BsNb PX4 could enhance the anti-tumour immune response by interfering with the formulation of the stroma and relieving the negative signal at the tumour site.

In conclusion, the bispecific nanobody binds to two different antigens and simultaneously blocks both pathways to achieve antitumour effects, possibly due to the recruitment of CTLs. However, the results obtained from the in vivo model conducted with cancer cell line-derived xenograft (CDX) might not fully represent the results obtained with patient-derived xenografts (PDX). Despite this difference, the present study indicated that the use of nanobodies could facilitate anti-cancer therapies, and nanobody-based drugs may have great potential in anti-cancer treatment.

## Conclusion

We designed a novel bispecific nanobody (BsNb PX4) targeting PD-L1 and CXCR4, which are involved in the metastasis and progression of pancreatic cancer. BsNb PX4 was characterized specifically in association with both cell surface antigens. The nanobody reduced the viability of tumour cells at a low dose, inhibited CXCL12-induced tumour migration and enhanced the cytotoxicity of hPBMCs in vitro. Furthermore, BsNb PX4 induced marked inhibition of human pancreatic tumour cell growth in NOD/SCID mice in vivo. A new approach to preclinically treat pancreatic cancer through an immune-therapeutic agent will be proposed following this project.

## Electronic supplementary material

Below is the link to the electronic supplementary material.

Supplementary Material 1

## Data Availability

The mass spectrometry proteomics data have been deposited to the ProteomeXchange Consortium via the PRIDE partner repository with the dataset identifier PXD037102.

## Abbreviations

PDAC: pancreatic ductal adenocarcinoma
CTLA-4: cytotoxic T-lymphocyte-associated protein 4
PD-1: programmed cell death-1
PD-L1: programmed death-ligand 1
TME: tumour microenvironment
CTLs: cytotoxic T lymphocytes
CRC: fibroblast activation protein
CRC: carcinoma of colon and rectum
CXCL12: chemokine (C-X-C motif) ligand 12
SDF-1: stromal cell-derived factor 1
sdAbs: Single-domain antibodies
VHH: variable domain of the heavy chain of HCAbs
HCAbs: heavy-chain antibodies
aTTP: Acquired Thrombotic Thrombocytopenic Purpura
IL-2: interleukin-2
FBS: fetal bovine serum
mAb: monoclonal antibody
hPBMCs: human peripheral blood mononuclear cell
PBS: phosphate buffer saline
SDS-PAGE: sodium dodecyl sulfate-polyacrylamide gel electrophoresis
ECM: extracellular matrix
MDSCs: myeloid-derived suppressor cells
Tregs: regulatory T cells
TAMs: tumour-associated macrophages
CAFs: cancer-associated fibroblasts
HCC: hepatocellular carcinoma
αSMA: Smooth muscle alpha-actin
CFSE: 5,6-carboxyfluorescein diacetate succinimide ester

## References

[1] Siegel RL, Miller KD, Jemal A, Cancer statistics, 2015. CA Cancer J Clin. 2015;65(1):5–29. 10.3322/caac.21254 25559415

[2] Li C (2014). Knockdown of ribosomal protein L39 by RNA interference inhibits the growth of human pancreatic cancer cells in vitro and in vivo. Biotechnol J.

[3] Li C (2020). RPL21 siRNA Blocks Proliferation in Pancreatic Cancer Cells by Inhibiting DNA Replication and Inducing G1 Arrest and Apoptosis. Front Oncol.

[4] Xiang XS (2022). Histone deacetylases: A novel class of therapeutic targets for pancreatic cancer. Biochim Biophys Acta Rev Cancer.

[5] Sarantis P (2020). Pancreatic ductal adenocarcinoma: Treatment hurdles, tumor microenvironment and immunotherapy. World J Gastrointest Oncol.

[6] Biankin AV, Maitra A (2015). Subtyping Pancreat Cancer Cancer Cell.

[7] Collins JM, Redman JM, Gulley JL (2018). Combining vaccines and immune checkpoint inhibitors to prime, expand, and facilitate effective tumor immunotherapy. Expert Rev Vaccines.

[8] Tanaka A, Sakaguchi S (2019). Targeting Treg cells in cancer immunotherapy. Eur J Immunol.

[9] Bommareddy PK, Shettigar M, Kaufman HL (2018). Integrating oncolytic viruses in combination cancer immunotherapy. Nat Rev Immunol.

[10] Kazandjian D (2016). FDA Approval Summary: Nivolumab for the Treatment of Metastatic Non-Small Cell Lung Cancer With Progression On or After Platinum-Based Chemotherapy. Oncologist.

[11] Di Giacomo AM (2012). Ipilimumab and fotemustine in patients with advanced melanoma (NIBIT-M1): an open-label, single-arm phase 2 trial. Lancet Oncol.

[12] Brahmer JR (2012). Safety and activity of anti-PD-L1 antibody in patients with advanced cancer. N Engl J Med.

[13] Royal RE (2010). Phase 2 trial of single agent Ipilimumab (anti-CTLA-4) for locally advanced or metastatic pancreatic adenocarcinoma. J Immunother.

[14] Joyce JA, Fearon DT (2015). T cell exclusion, immune privilege, and the tumor microenvironment. Science.

[15] Feig C (2013). Targeting CXCL12 from FAP-expressing carcinoma-associated fibroblasts synergizes with anti-PD-L1 immunotherapy in pancreatic cancer. Proc Natl Acad Sci U S A.

[16] Fearon DT (2014). The carcinoma-associated fibroblast expressing fibroblast activation protein and escape from immune surveillance. Cancer Immunol Res.

[17] Righi E (2011). CXCL12/CXCR4 blockade induces multimodal antitumor effects that prolong survival in an immunocompetent mouse model of ovarian cancer. Cancer Res.

[18] Cortez-Retamozo V (2002). Efficient tumor targeting by single-domain antibody fragments of camels. Int J Cancer.

[19] Abulrob A (2005). The blood-brain barrier transmigrating single domain antibody: mechanisms of transport and antigenic epitopes in human brain endothelial cells. J Neurochem.

[20] Salvador JP, Vilaplana L, Marco MP (2019). Nanobody: outstanding features for diagnostic and therapeutic applications. Anal Bioanal Chem.

[21] Scully M (2019). Caplacizumab Treatment for Acquired Thrombotic Thrombocytopenic Purpura. N Engl J Med.

[22] Steeland S, Vandenbroucke RE, Libert C (2016). Nanobodies as therapeutics: big opportunities for small antibodies. Drug Discov Today.

[23] Yang EY, Shah K (2020). Nanobodies: Next Generation of Cancer Diagnostics and Therapeutics. Front Oncol.

[24] Liu X (2020). Construction of Novel Bispecific Single-Domain Antibodies (BiSdAbs) with Potent Antiangiogenic Activities. Pharm Fronts.

[25] Zhou Y (2020). A novel bispecific antibody targeting CD3 and prolactin receptor (PRLR) against PRLR-expression breast cancer. J Exp Clin Cancer Res.

[26] Sun R, et al., A Rational Designed Novel Bispecific Antibody for the Treatment of GBM. Biomedicines. 2021;9(6). 10.3390/biomedicines9060640 PMC8230177 34204931

[27] Chen J, et al., A Novel Bispecific Antibody Targeting CD3 and Lewis Y with Potent Therapeutic Efficacy against Gastric Cancer. Biomedicines. 2021;9(8). 10.3390/biomedicines9081059 PMC8393954 34440263

[28] Ge Q (2020). Generating a Novel Bispecific Nanobody to Enhance Antitumor Activity. Pharm Fronts.

[29] Zhang F (2017). Structural basis of a novel PD-L1 nanobody for immune checkpoint blockade. Cell Discov.

[30] Ostrand-Rosenberg S, Fenselau C (2018). Myeloid-Derived Suppressor Cells: Immune-Suppressive Cells That Impair Antitumor Immunity and Are Sculpted by Their Environment. J Immunol.

[31] Gabrilovich DI, Ostrand-Rosenberg S, Bronte V (2012). Coordinated regulation of myeloid cells by tumours. Nat Rev Immunol.

[32] Karakhanova S (2015). Characterization of myeloid leukocytes and soluble mediators in pancreatic cancer: importance of myeloid-derived suppressor cells. Oncoimmunology.

[33] Iwai Y (2017). Cancer immunotherapies targeting the PD-1 signaling pathway. J Biomed Sci.

[34] Siret C (2019). Deciphering the Crosstalk Between Myeloid-Derived Suppressor Cells and Regulatory T Cells in Pancreatic Ductal Adenocarcinoma. Front Immunol.

[35] Huang B (2006). Gr-1 + CD115 + immature myeloid suppressor cells mediate the development of tumor-induced T regulatory cells and T-cell anergy in tumor-bearing host. Cancer Res.

[36] Feng M (2017). PD-1/PD-L1 and immunotherapy for pancreatic cancer. Cancer Lett.

[37] Norton J, et al., Pancreatic Cancer Associated Fibroblasts (CAF): Under-Explored Target for Pancreatic Cancer Treatment. Cancers (Basel). 2020;12(5). 10.3390/cancers12051347 PMC7281461 32466266

[38] Binenbaum Y, Na’ara S, Gil Z (2015). Gemcitabine resistance in pancreatic ductal adenocarcinoma. Drug Resist Updat.

[39] Whatcott CJ (2015). Desmoplasia in Primary Tumors and Metastatic Lesions of Pancreatic Cancer. Clin Cancer Res.

[40] Domanska UM (2012). CXCR4 inhibition with AMD3100 sensitizes prostate cancer to docetaxel chemotherapy. Neoplasia.

[41] Orimo A (2005). Stromal fibroblasts present in invasive human breast carcinomas promote tumor growth and angiogenesis through elevated SDF-1/CXCL12 secretion. Cell.

[42] Chen Y (2015). CXCR4 inhibition in tumor microenvironment facilitates anti-programmed death receptor-1 immunotherapy in sorafenib-treated hepatocellular carcinoma in mice. Hepatology.

[43] Bockorny B (2020). BL-8040, a CXCR4 antagonist, in combination with pembrolizumab and chemotherapy for pancreatic cancer: the COMBAT trial. Nat Med.

[44] Xian Z (2019). Blocking the PD-1-PD-L1 axis by a novel PD-1 specific nanobody expressed in yeast as a potential therapeutic for immunotherapy. Biochem Biophys Res Commun.

[45] Li J (2021). Subcutaneous envafolimab monotherapy in patients with advanced defective mismatch repair/microsatellite instability high solid tumors. J Hematol Oncol.

[46] Markham A (2022). Envafolimab: First Approval. Drugs.

[47] Papadopoulos KP (2021). First-in-Human Phase I Study of Envafolimab, a Novel Subcutaneous Single-Domain Anti-PD-L1 Antibody, in Patients with Advanced Solid Tumors. Oncologist.

[48] Vela M (2015). Chemokine receptor-specific antibodies in cancer immunotherapy: achievements and challenges. Front Immunol.

[49] Jähnichen S (2010). CXCR4 nanobodies (VHH-based single variable domains) potently inhibit chemotaxis and HIV-1 replication and mobilize stem cells. Proc Natl Acad Sci U S A.

[50] Bobkov V (2018). Nanobody-Fc constructs targeting chemokine receptor CXCR4 potently inhibit signaling and CXCR4-mediated HIV-entry and induce antibody effector functions. Biochem Pharmacol.

[51] Fujita H (2010). alpha-Smooth Muscle Actin Expressing Stroma Promotes an Aggressive Tumor Biology in Pancreatic Ductal Adenocarcinoma. Pancreas.

