# Supplementary material for: Tumour inhibitory activity on pancreatic cancer by bispecific nanobody targeting PD-L1 and CXCR4
Source: BMC Cancer. 2022 Oct 25;22:1092. doi: 10.1186/s12885-022-10165-7 (PMC9594910; doi:10.1186/s12885-022-10165-7)
Supplement: Supplementary file 1 — Supplementary Material 1 [file 12885_2022_10165_MOESM1_ESM.pptx]

## Slide 1
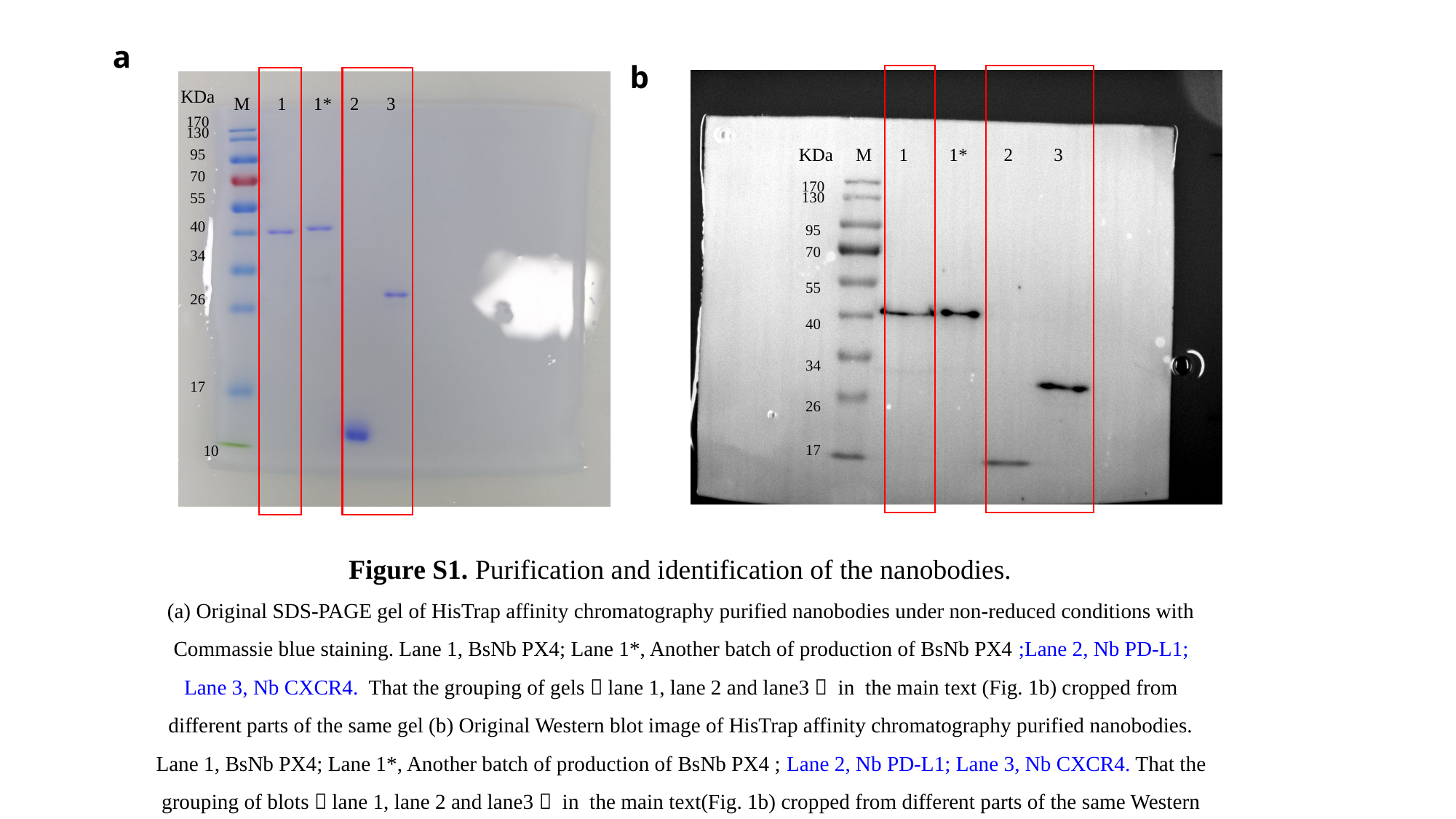

a
b
 KDa
 170 130
 95
 70
 55
 40
 34
 26
 17
 10
 M 1 1* 2 3
 KDa M 1 1* 2 3
 170 130
 95
 70
 55
 40
 34
 26
 17
 Figure S1. Purification and identification of the nanobodies.
(a) Original SDS-PAGE gel of HisTrap affinity chromatography purified nanobodies under non-reduced conditions with Commassie blue staining. Lane 1, BsNb PX4; Lane 1*, Another batch of production of BsNb PX4 ;Lane 2, Nb PD-L1; Lane 3, Nb CXCR4. That the grouping of gels（lane 1, lane 2 and lane3） in the main text (Fig. 1b) cropped from different parts of the same gel (b) Original Western blot image of HisTrap affinity chromatography purified nanobodies. Lane 1, BsNb PX4; Lane 1*, Another batch of production of BsNb PX4 ; Lane 2, Nb PD-L1; Lane 3, Nb CXCR4. That the grouping of blots（lane 1, lane 2 and lane3） in the main text(Fig. 1b) cropped from different parts of the same Western blot image.
